# Cooking Coal Use and All-Cause and Cause-Specific Mortality in a Prospective Cohort Study of Women in Shanghai, China

**DOI:** 10.1289/EHP236

**Published:** 2016-04-19

**Authors:** Christopher Kim, Wei Jie Seow, Xiao-Ou Shu, Bryan A. Bassig, Nathaniel Rothman, Bingshu E. Chen, Yong-Bing Xiang, H. Dean Hosgood, Bu-Tian Ji, Wei Hu, Cuiju Wen, Wong-Ho Chow, Qiuyin Cai, Gong Yang, Yu-Tang Gao, Wei Zheng, Qing Lan

**Affiliations:** 1 Division of Cancer Epidemiology and Genetics, National Cancer Institute, National Institutes of Health, Department of Health and Human Services, Rockville, Maryland, USA; 2 Vanderbilt University Medical Center, Nashville, Tennessee, USA; 3 Queen’s Cancer Research Institute, Queen’s University, Kingston, Ontario, Canada; 4 Shanghai Cancer Institute, Renji Hospital, Shanghai Jiaotong University School of Medicine, Shanghai, China; 5 Department of Epidemiology and Population Health, Albert Einstein College of Medicine, New York, New York, USA; 6 Department of Epidemiology, University of Texas MD Anderson Cancer Center, Houston, Texas, USA

## Abstract

**Background:**

Nearly 4.3 million deaths worldwide were attributable to exposure to household air pollution in 2012. However, household coal use remains widespread.

**Objectives:**

We investigated the association of cooking coal and all-cause and cause-specific mortality in a prospective cohort of primarily never-smoking women in Shanghai, China.

**Methods:**

A cohort of 74,941 women were followed from 1996 through 2009 with annual linkage to the Shanghai vital statistics database. Cause-specific mortality was identified through 2009. Use of household coal for cooking was assessed through a residential history questionnaire. Cox proportional hazards models estimated the risk of mortality associated with household coal use.

**Results:**

In this cohort, 63% of the women ever used coal (n = 46,287). Compared with never coal use, ever use of coal was associated with mortality from all causes [hazard ratio (HR) = 1.12; 95% confidence interval (CI): 1.05, 1.21], cancer (HR = 1.14; 95% CI: 1.03, 1.27), and ischemic heart disease (overall HR = 1.61; 95% CI: 1.14, 2.27; HR for myocardial infarction specifically = 1.80; 95% CI: 1.16, 2.79). The risk of cardiovascular mortality increased with increasing duration of coal use, compared with the risk in never users. The association between coal use and ischemic heart disease mortality diminished with increasing years since cessation of coal use.

**Conclusions:**

Evidence from this study suggests that past use of coal among women in Shanghai is associated with excess all-cause mortality, and from cardiovascular diseases in particular. The decreasing association with cardiovascular mortality as the time since last use of coal increased emphasizes the importance of reducing use of household coal where use is still widespread.

**Citation:**

Kim C, Seow WJ, Shu XO, Bassig BA, Rothman N, Chen BE, Xiang YB, Hosgood HD III, Ji BT, Hu W, Wen C, Chow WH, Cai Q, Yang G, Gao YT, Zheng W, Lan Q. 2016. Cooking coal use and all-cause and cause-specific mortality in a prospective cohort study of women in Shanghai, China. Environ Health Perspect 124:1384–1389; http://dx.doi.org/10.1289/EHP236

## Introduction

An estimated 3 billion people worldwide burn solid fuels in simple stoves or pits for cooking and heating in their homes (UNDP and WHO 2009). The majority of these individuals live in low- to middle-income countries. Before the rapid economic development of urban centers in China, solid fuels were the predominant fuel source for household heating and cooking and are currently used in rural parts of the country (IARC 2010; Zhang and Smith 2007). As a result, hazardous indoor levels of breathable pollutants such as particulate matter (PM) could result due to poor household air quality (Zhou et al. 2011).

Household air pollution (HAP) was estimated to account for approximately 3.9 million premature deaths (Lim et al. 2012; Smith et al. 2014), 4.3% of lost healthy years, > 100,000 disability-adjusted life years in 2010 (Lim et al. 2012), and nearly 4.3 million deaths worldwide in 2012 (WHO 2014). Globally, HAP was the third leading cause of disease burden in 2010 (Lim et al. 2012) and has been associated with a variety of morbidities, including cancer, cardiovascular disease, cataracts, low birth weight, and pulmonary diseases (Kim et al. 2011). In populations with high levels of exposure, the risk of developing lung cancer can be increased by ≥ 10-fold compared with never-users of coal/solid fuel (Armstrong et al. 2004; Barone-Adesi et al. 2012; Kim et al. 2011; Lan et al. 2002). Even in modern-day East Asia, HAP is among the top five risk factors for disease and mortality (Lim et al. 2012). Much of the burden in this region occurs in rural China, where an estimated 420,000–700,000 premature deaths due to HAP occur annually (Mestl et al. 2007; Zhang and Smith 2007). An estimated 5.5 years of average life lost in China from cardiovascular-related mortality was attributable to ambient total suspended particulates (Chen et al. 2013).

Previous studies in China have estimated associations between solid fuel use and various diseases (Kim et al. 2011; Zhang et al. 1988). In Xuanwei, China, a region with heavy household coal use, lung cancer incidence/mortality, chronic obstructive pulmonary disease incidence, and pneumonia mortality have been elevated (Barone-Adesi et al. 2012; Chapman et al. 2005; Lan et al. 2002; Lee et al. 2010; Shen et al. 2009); however, these studies have been conducted in rural and underdeveloped populations with active household coal use. Rapid development has improved the quality of housing over time, leading to reduced HAP exposure (IARC 2010). A recent cross-sectional study in urban Shanghai, China, found an increased prevalence of hypertension, cardiovascular heart disease, stroke, and diabetes among adults who used solid fuels (Lee et al. 2012). However, no prospective study has evaluated whether historic coal exposures are associated with risk of mortality years after HAP levels decreased in an urban setting among never-smoking women.

Because current use of coal is substantially lower than it once was in Shanghai, little research has been done on the residual health risks associated with latency of health effects due to household coal use. Here, the association between past coal use and mortality (total and cause-specific) among actively followed participants in the prospective Shanghai Women’s Health Study was assessed.

## Methods

### Study Population

The Shanghai Women’s Health Study cohort has been described in detail (Zheng et al. 2005). Briefly, a list of residents was obtained from municipal offices for 81,170 women, ages 40–70 years, in seven communities located in urban Shanghai, China. A total of 75,221 (92.7%) women participated in the study and completed baseline surveys between 1996 and 2000. Of these, 74,941 women were followed through December 2009 with surveys and periodic linkage to cancer and vital statistics registries. After excluding women who were found to be < 40 years or > 70 years of age, and/or had prevalent cancer, the remaining cohort consisted of 73,363 women. We evaluated mortality from all causes [*International Classification of Diseases, 9th Revision* (ICD-9) codes: 001–999] as well as cause-specific mortality from all cancer (ICD-9: 140–208), lung cancer (ICD-9: 162), all cardiovascular diseases (ICD-9: 390–459), all gastrointestinal diseases (ICD-9: 520–579), all kidney diseases (ICD-9: 580–593), and diabetes (ICD-9: 250). We further conducted analyses for more specific causes of cardiovascular disease mortality, including ischemic heart disease (ICD-9: 410–414), myocardial infarction (ICD-9: 410), overall stroke (ICD-9: 430–438), ischemic stroke (ICD-9: 434), and hemorrhagic stroke (ICD-9: 430–432). Women were excluded from analyses of specific causes of death if they reported a history of corresponding diseases at baseline, such that women with a baseline history of coronary heart disease were excluded from analyses of cardiovascular mortality. Similarly, women with a baseline history of stroke, diabetes, or gastrointestinal diseases (chronic gastritis, pancreatitis, hepatitis, intestinal polyp, or ulcerative colitis) were excluded from analyses of stroke, diabetes, and gastrointestinal mortality, respectively. Kidney disease history was not assessed at baseline, so women with prevalent kidney disease could not be excluded from analyses of mortality due to kidney disease. A total of 3,808 deaths were identified through 2009. All participants gave informed consent, and the protocols used were approved by the institutional review boards of all collaborative institutions.

### Data Collection and Follow-up

Information on household residences was collected in the baseline survey (1996–2000) that used a standardized questionnaire and in-person interview. Each subject was asked about current residence and two past residences in which the subject lived, years moved in and out, cooking fuel used (gas, coal), and kitchen ventilation (good, fairly good, or poor). From this information, ever use of coal (defined as > 1 year coal use), years of coal use, time period of coal use, and poor ventilation (ever or never) were determined. Additional information on medical history, household cohabitant habits, employment, demographics, lifestyle, and diet were collected at baseline. Vital status and cause of death (ICD-9) were ascertained from death certificate linkage to the Shanghai Vital Statistics Unit through 2009.

### Statistical Analysis

Cox proportional hazards regression models were used to estimate the relative risk of mortality for coal use and are presented as hazard ratios (HRs) with 95% confidence intervals (CIs). The time scale was person-years, which was based on the date at death or the date at the end of follow-up, whichever event came first. All models were stratified by birth cohort (1930s, 1940s, > 1950). Most (99%) of the women had stopped using coal before they enrolled in the cohort. Coal usage was calculated up through the most recent available information (baseline interview date for household conditions) and held fixed through the end of follow-up. Coal use was evaluated as a non–time-dependent variable in models as the main effect for ever coal use (dichotomous; ever or never) and duration of time used (categorical; > 0–15, > 15–30, > 30 years), and as a time-dependent variable for years since last use (categorical; > 0–10, > 10–20, > 20 years) compared with women who never used coal, who served as the reference group. To calculate *p*-trends, the coal variables were included in the models as ordinal variables and were modeled continuously, with the Wald chi-square values used to assess statistical significance. To test the proportional hazards assumption, the interaction term for ever/never coal use and the natural log of age from enrollment were tested; *p*-values were > 0.05, consistent with the assumption of proportional hazards (data not shown). Adjustment for covariates included age (continuous), smoking status (≥ 1 cigarette/day for ≥ 6 months; ever/never), family income (< 20,000 yuan/year, ≥ 20,000 yuan/year), environmental tobacco smoke (ever/never), occupational history (technical, governmental, administrative, manufacturing), education (college and above, high school, middle school, elementary school or less), shift work (ever or never), body mass index (BMI; continuous), postmenopausal hormone replacement therapy [HRT; ever or never (no HRT or premenopausal HRT)], parity (number of births), regular alcohol consumption (3 drinks/week for ≥ 6 months, ever or never), marital status (single, married, separated/divorced, widowed), total caloric intake (kcals/day; continuous), poor kitchen ventilation (ever or never), and leisure time physical activity (hours/week/year, continuous). All covariates were defined at baseline. We included a missing data category for categorical variables. Missing data for continuous covariates were excluded in the analysis. Women who died of accidents or trauma (ICD-9: E800–999) were not distributed substantially differently between coal users and non-coal users, and exclusion of these women did not alter the overall results; thus, these causes of death were included in the final analyses (data not shown). To check for residual confounding, we performed sensitivity analyses by stratifying the analyses by socioeconomic factors (family annual income, education, kitchen ventilation) that were significantly associated with all-cause mortality in univariate analyses and that had sufficient numbers (> 5%) of coal users in each category. We also used separate models to test for possible interactions between coal use and three indicators of socioeconomic status [education (middle school and above or elementary school and below), kitchen ventilation (always good or not good), and family annual income (above or below 20,000 yuan)] by modeling interaction terms. All analyses were performed in SAS 9.3 with the PHREG procedure (SAS Institute Inc., Cary, NC), and a *p*-value of < 0.05 was considered statistically significant.

## Results

Distributions of various characteristics in the cohort by coal use are presented in Table 1. About 63% of the women in this population ever used coal (median duration among coal users, 24 years). Very few women were ever smokers, and most were exposed to environmental tobacco smoke. Coal users were more likely to have less education, worked in manufacturing, ever had a night shift job, and earned less income than did never coal users. At baseline, 1.1% (*n* = 836) of the population still used coal.

**Table 1:** Baseline characteristics and mortality and follow-up of the Shanghai Women’s Health Cohort, 1996–2000, by ever coal use [mean ± SD or number (%) unless otherwise indicated].

Characteristics	Never used coal (*n *= 27,076) [mean ± SD or *n* (%)]	Ever used coal (*n *= 46,287) [mean ± SD or *n* (%)]	All-cause mortality^*a*^ [HR (95% CI)]
Age at baseline (years)	51.97 ± 8.88	52.05 ± 9.16	—
Birth cohort			—
< 1940	7,939 (29)	13,733 (30)
1940–1949	7,609 (28)	11,711 (25)
≥ 1950	11,528 (43)	20,843 (45)
Education
Elementary school and less	4,581 (17)	11,106 (24)	1.00 (reference)
Middle school	8,654 (32)	18,616 (40)	0.65 (0.59, 0.71)
High school	8,377 (31)	12,113 (26)	0.53 (0.48, 0.58)
College and above	5,456 (20)	4,447 (10)	0.40 (0.35, 0.46)
Unknown	8	5	—
Annual family income
≥ Median (≥ 20,000 yuan)	14,324 (53)	19,147 (41)	1.00 (reference)
< Median (< 20,000 yuan)	12,742 (47)	27,134 (59)	1.43 (1.33, 1.53)
Unknown	10	6	—
Ever smoker
No	26,462 (98)	44,858 (97)	1.00 (reference)
Yes	614 (2)	1,429 (3)	1.81 (1.60, 2.04)
Ever ETS at follow-up (for nonsmokers only)
Yes	18,191 (77)	32,118 (77)	1.00 (reference)
No	5,557 (23)	9,360 (23)	1.08 (1.00, 1.17)
Unknown	2	1	—
Alcohol consumption (≥ 3 drinks/week; ≥ 6 months)	
No	26,428 (98)	45,009 (97)	1.00 (reference)
Yes	546 (2)	1,104 (3)	1.00 (0.81, 1.22)
Unknown	102	174	—
Marital status
Single	257 (1)	378 (1)	1.00 (reference)
Married	24,058 (89)	41,102 (89)	0.65 (0.45, 0.94)
Separated/divorced	864 (3)	1,285 (3)	0.76 (0.50, 1.15)
Widowed	1,897 (7)	3,522 (7)	0.92 (0.63, 1.33)
Number of pregnancies
0	774 (3)	1,185 (2)	1.07 (1.04, 1.10)
1	4,734 (17)	7,029 (15)
2	8,050 (30)	13,442 (29)
3	6,568 (24)	11,096 (24)
4	3,958 (15)	7,202 (16)
≥ 5	2,992 (11)	6,333 (14)
Hormone replacement therapy (HRT)
Never/premenopausal HRT	25,930 (96)	44,838 (97)	1.00 (reference)
Ever	1,146 (4)	1,449 (3)	0.53 (0.40, 0.69)
Occupation^*b*^
Production and Manufacturing Workers	11,494 (43)	25,368 (55)	1.00 (reference)
Technicians and Professionals	8,794 (33)	9,552 (21)	0.58 (0.53, 0.63)
Government, Political, and Legal Workers	1,198 (4)	1,482 (3)	0.62 (0.51, 0.75)
Administrative and Service Workers	5,486 (20)	9,712 (21)	0.95 (0.88, 1.03)
Never worked	101 (0)	173 (0)
Unknown	3	0	—
Night shift job
Never	20,010 (77)	32,230 (72)	1.00 (reference)
Ever	6,081 (23)	12,487 (28)	1.04 (0.96, 1.13)
Unknown	985	1,570	—
BMI (kg/m^2^)	23.81 ± 3.35	24.13 ± 3.46
Median (25th–75th percentile)	23.53 (21.48–25.81)	23.83 (21.72–26.22)
< Median	13,503 (50)	21,474 (46)	1.00 (reference)
≥ Median	13,573 (50)	24,813 (54)	1.03 (0.97–1.10)
Unknown	28	31
Caloric intake (kcal/day)	1702.39 ± 339.06	1675.01 ± 412.03
Median (25th–75th percentile)	1661.22 (1433.03–1919.38)	1631.56 (1398.10–1902.63)
< Median	13,538 (50)	24,585 (53)	1.00 (reference)
≥ Median	13,538 (50)	21,702 (47)	0.80 (0.75–0.85)
Physical activity (MET-hr/week/year)	103.09 ± 44.61	108.58 ± 45.37
Median (25th–75th percentile)	96.20 (71.15–127.49)	102.23 (76.65–133.76)
< Median	13,540 (50)	20,394 (44)	1.00 (reference)
≥ Median	13,536 (50)	25,893 (56)	0.79 (0.74–0.84)
Coal use (years)		23.37 ± 13.46
Median (25th–75th percentile)	0	24 (12–33)
< Median	0	21,977 (47)	1.00 (reference)
≥ Median	0	24,310 (53)	1.24 (1.16–1.32)
Ever poor ventilation
No	24,903 (92)	33,845 (73)	1.00 (reference)
Yes	2,173 (8)	12,442 (27)	1.15 (1.07, 1.24)
History of coronary heart disease
No	25,073 (93)	42,937 (93)	1.00 (reference)
Yes	2,003 (7)	3,350 (7)	1.32 (1.21, 1.45)
History of stroke
No	26,767 (99)	45,741 (99)	1.00 (reference)
Yes	309 (1)	546 (1)	3.28 (2.88, 3.73)
History of diabetes
No	26,023 (96)	44,169 (95)	1.00 (reference)
Yes	1,053 (4)	2,118 (5)	2.55 (2.34, 2.78)
History of gastrointestinal diseases^*c*^
No	20,754 (77)	36,306 (78)	1.00 (reference)
Yes	6,322 (23)	9,981 (22)	0.90 (0.83, 0.97)
Abbreviations: ETS, environmental tobacco smoke; MET, metabolic equivalent. Data are complete for all women unless otherwise noted. ^***a***^Univariate associations with all-cause mortality, stratified by birth cohort. ^***b***^Each occupation was coded according to the Chinese National Standard Occupation and Industry Codes Manual. Occupations were defined as Technicians and Professionals (010–149); Government, Political, and Legal Workers (150–249); Administrative and Service Workers (310–599); Production and Manufacturing Workers (600–998) (Chinese National Bureau of Statistics, General Administration of National Standards, Office of the National Census of the State Council 1982). ^***c***^Includes chronic gastritis, pancreatitis, hepatitis, intestinal polyp, or ulcerative colitis.			

The association of coal use for cooking and various causes of mortality are presented in Table 2. A total of 3,808 deaths were identified through December 2009; of these, 2,500 individuals ever used coal. We estimated significant positive associations between ever (vs. never) coal use and all-cause mortality (HR = 1.12; 95% CI: 1.05, 1.21) and cancer-related mortality (HR = 1.14; 95% CI: 1.03, 1.27). Ever coal use was also associated with overall cardiovascular mortality (HR = 1.18; 95% CI: 1.02, 1.37), and more specifically with ischemic heart disease (HR = 1.61; 95% CI: 1.14, 2.27), including myocardial infarction (HR = 1.80; 95% CI: 1.16, 2.79). However, ever use of coal was not associated with stroke mortality, or with deaths due to ischemic or hemorrhagic stroke specifically. We stratified the analysis by education, family annual income, and kitchen ventilation, and these analyses did not appreciably change the associations (see Tables S1–S3). Further, none of the interaction terms were significant for any mortality outcome (see Tables S1–S3).

**Table 2:** Adjusted hazard ratios for all-cause and cause-specific mortality by ever usage of coal in Shanghai Women’s Health Study.

Cause of death (ICD-9 codes)	Never used coal		Ever used coal	
	Deaths^*a*^	HR (95% CI)^*b*^	Deaths^*a*^	HR (95% CI)^*b*^
All-cause	1,308	1.00 (reference)	2,500	1.12 (1.05, 1.21)
Cancer (140–208)	575	1.00 (reference)	1,045	1.14 (1.03, 1.27)
Lung cancer (162)	117	1.00 (reference)	215	1.20 (0.95, 1.52)
Cardiovascular (390–459)	276	1.00 (reference)	583	1.18 (1.02, 1.37)
Ischemic heart disease (410–414)	47	1.00 (reference)	133	1.61 (1.14, 2.27)
Myocardial infarction (410)	29	1.00 (reference)	91	1.80 (1.16, 2.79)
Stroke (430–438)	185	1.00 (reference)	349	1.01 (0.84, 1.22)
Ischemic stroke (434)	74	1.00 (reference)	145	1.08 (0.80, 1.45)
Hemorrhagic stroke (430–432)	99	1.00 (reference)	165	0.91 (0.70, 1.19)
Gastrointestinal (520–579)	26	1.00 (reference)	55	1.24 (0.75, 2.04)
Kidney disease (580–593)	25	1.00 (reference)	46	1.11 (0.66, 1.84)
Diabetes (250)	23	1.00 (reference)	66	1.37 (0.83, 2.26)
^***a***^Women were excluded from analyses of specific causes of death if they reported a history of corresponding diseases at baseline. ^***b***^Adjusted for age, smoking status, environmental tobacco smoke, occupation, education, shift work, BMI, hormone replacement therapy, family income, parity, alcohol drinking, marital status, caloric intake, physical activity, and stove ventilation.				

Duration of coal use and causes of mortality are described in Table 3. Compared with never coal users, additional years of coal use were associated with a monotonic increase in the risk of mortality from cardiovascular disease [> 0–15 years: HR = 1.07 (95% CI: 0.86, 1.32); > 15–30 years: HR = 1.08 (95% CI: 0.89, 1.32); > 30 years: HR = 1.32 (95% CI: 1.11, 1.57) *p*-trend = 0.0023], specifically for ischemic heart disease [> 0–15 years: HR = 1.25 (95% CI: 0.76, 2.05); > 15–30 years: HR = 1.46 (95% CI: 0.94, 2.27); > 30 years: HR = 1.91 (95% CI: 1.30, 2.80) *p*-trend = 0.00080], including myocardial infarction [> 0–15 years: HR = 1.43 (95% CI: 0.78, 2.62); > 15–30 years: HR = 1.86 (95% CI: 1.09, 3.16); > 30 years: HR = 1.98 (95% CI: 1.21, 3.22) *p*-trend = 0.0044]. Women had an elevated risk of all-cause mortality across all three coal use duration categories [> 0–15 years: HR = 1.13 (95% CI: 1.02, 1.24); > 15–30 years: HR = 1.10 (95% CI: 1.00, 1.20); > 30 years: HR = 1.14 (95% CI: 1.05, 1.25) *p*-trend = 0.0035] compared with never users of coal.

**Table 3:** Adjusted hazard ratios for all-cause and cause-specific mortality by years of coal use in Shanghai Women’s Health Study.

Cause of death (ICD-9 codes)	Never		> 0–15 years		> 15–30 years		> 30 years		*p*-Trend^*c*^
	Deaths^*a*^	HR (95% CI)^*b*^	Deaths^*a*^	HR (95% CI)^*b*^	Deaths^*a*^	HR (95% CI)^*b*^	Deaths^*a*^	HR (95% CI)^*b*^	
All-cause	1,308	1.00 (reference)	638	1.13 (1.02, 1.24)	790	1.10 (1.00, 1.20)	1,072	1.14 (1.05, 1.25)	0.0035
Cancer (140–208)	575	1.00 (reference)	290	1.18 (1.02, 1.37)	359	1.19 (1.04, 1.36)	396	1.08 (0.95, 1.24)	0.16
Lung cancer (162)	117	1.00 (reference)	56	1.18 (0.85, 1.63)	79	1.34 (1.00, 1.80)	80	1.09 (0.81, 1.47)	0.34
Cardiovascular (390–459)	276	1.00 (reference)	130	1.07 (0.86, 1.32)	167	1.08 (0.89, 1.32)	286	1.32 (1.11, 1.57)	0.0023
Ischemic heart disease (410–414)	47	1.00 (reference)	25	1.25 (0.76, 2.05)	37	1.46 (0.94, 2.27)	71	1.91 (1.30, 2.80)	0.0008
Myocardial infarction (410)	29	1.00 (reference)	18	1.43 (0.78, 2.62)	29	1.86 (1.09, 3.16)	44	1.98 (1.21, 3.22)	0.0044
Stroke (430–438)	185	1.00 (reference)	85	1.03 (0.79, 1.34)	105	0.95 (0.74, 1.22)	159	1.05 (0.84, 1.31)	0.82
Ischemic stroke (434)	74	1.00 (reference)	29	0.92 (0.59, 1.43)	49	1.17 (0.81, 1.70)	67	1.10 (0.77, 1.55)	0.45
Hemorrhagic stroke (430–432)	99	1.00 (reference)	47	1.04 (0.73, 1.50)	46	0.77 (0.53, 1.10)	72	0.94 (0.68, 1.30)	0.43
Gastrointestinal (520–579)	26	1.00 (reference)	13	1.21 (0.61, 2.41)	20	1.38 (0.76, 2.54)	22	1.14 (0.63, 2.08)	0.57
Kidney disease (580–593)	25	1.00 (reference)	14	1.31 (0.67, 2.57)	11	0.82 (0.40, 1.69)	21	1.21 (0.66, 2.22)	0.78
Diabetes (250)	23	1.00 (reference)	14	1.22 (0.62, 2.43)	19	1.28 (0.68, 2.40)	33	1.51 (0.86, 2.64)	0.15
^***a***^Women were excluded from analyses of specific causes of death if they reported a history of corresponding diseases at baseline. ^***b***^Adjusted for age, smoking status, environmental tobacco smoke, occupation, education, shift work, BMI, hormone replacement therapy, family income, parity, alcohol drinking, marital status, caloric intake, physical activity, and stove ventilation. ^***c***^*p*-Trend calculated by treating categorical coal years as ordinal variables and modeling it continuously.									

Years since cessation of coal use from baseline and mortality risk are presented in Table 4. For women with > 0–10 years since last coal use, positive associations were observed for ischemic heart disease mortality (HR = 2.57; 95% CI: 1.39, 4.78), specifically myocardial infarction mortality (HR = 2.80; 95% CI: 1.37, 5.73). Relative risks of overall cardiovascular mortality, specifically mortality from ischemic heart disease including myocardial infarction, decreased monotonically over time (*p*-trend < 0.05) and were not significantly elevated in those with > 20 years since last coal use (ischemic heart disease HR = 1.20; 95% CI: 0.81, 1.78; myocardial infarction HR = 1.32; 95% CI: 0.80, 2.18). The *p*-trends for the other mortality end points were not significant.

**Table 4:** Adjusted hazard ratios for all-cause and cause-specific mortality by years since last coal use in the home in Shanghai Women’s Health Study, using time-dependent covariate for years since last coal use.

Cause of death (ICD-9 codes)	Never HR (95% CI)^*a,b*^	> 0–10 years HR (95% CI)^*a,b*^	> 10–20 years HR (95% CI)^*a,b*^	> 20 years HR (95% CI)^*a,b*^	*p*-Trend^*c*^
All-cause	1.00 (reference)	1.00 (0.85, 1.18)	1.05 (0.96, 1.15)	1.07 (0.99, 1.16)	0.42
Cancer (140–208)	1.00 (reference)	0.91 (0.70, 1.20)	1.04 (0.91, 1.19)	1.14 (1.01, 1.28)	0.89
Lung cancer (162)	1.00 (reference)	1.35 (0.80, 2.30)	1.09 (0.81, 1.47)	1.11 (0.85, 1.44)	0.33
Cardiovascular (390–459)	1.00 (reference)	1.19 (0.86, 1.64)	1.23 (1.03, 1.47)	1.01 (0.85, 1.20)	0.028
Ischemic heart disease (410–414)	1.00 (reference)	2.57 (1.39, 4.78)	1.67 (1.12, 2.50)	1.20 (0.81, 1.78)	0.00080
Myocardial infarction (410)	1.00 (reference)	2.80 (1.37, 5.73)	1.94 (1.18, 3.20)	1.32 (0.80, 2.18)	0.0010
Stroke (430–438)	1.00 (reference)	0.96 (0.62, 1.48)	0.97 (0.76, 1.22)	0.96 (0.78, 1.20)	0.76
Ischemic stroke (434)	1.00 (reference)	1.05 (0.51, 2.15)	0.97 (0.67, 1.41)	0.95 (0.69, 1.33)	0.96
Hemorrhagic stroke (430–432)	1.00 (reference)	0.92 (0.51, 1.65)	0.88 (0.63, 1.23)	0.92 (0.68, 1.26)	0.50
Gastrointestinal (520–579)	1.00 (reference)	1.05 (0.35, 3.14)	0.87 (0.46, 1.66)	1.38 (0.81, 2.37)	0.78
Kidney disease (580–593)	1.00 (reference)	0.69 (0.20, 2.36)	0.82 (0.41, 1.63)	1.40 (0.80, 2.43)	0.48
Diabetes (250)	1.00 (reference)	0.59 (0.14, 2.58)	1.30 (0.73, 2.34)	1.28 (0.74, 2.20)	0.76
^***a***^Women were excluded from analyses of specific causes of death if they reported a history of corresponding diseases at baseline. ^***b***^Adjusted for age, smoking status, environmental tobacco smoke, occupation, education, shift work, BMI, hormone replacement therapy, family income, parity, alcohol drinking, marital status, caloric intake, physical activity, and stove ventilation. ^***c***^*p*-Trend calculated by treating categorical years of coal use as ordinal variables and modeling it continuously.					

## Discussion

In this analysis of coal use and mortality in the prospective Shanghai Women’s Health Study, ever coal use during a woman’s lifetime was associated with an increased risk of all-cause mortality after adjusting for potential confounders. Coal use was also associated with overall cancer and cardiovascular disease mortality, and the association with cardiovascular disease mortality showed a clear duration–response relationship. In particular, longer duration of coal use (i.e., > 30 years) was associated with an increased risk of ischemic heart disease mortality, specifically for the myocardial infarction subtype. After cessation of coal use, the strength of the coal–mortality association weakened with increasing time since last coal use for cardiovascular disease mortality. To the best of our knowledge, this is the first prospective study of coal use and mortality in an urban setting among never-smoking women in Asia with longitudinal vital status assessment. Additionally, we believe this is the first report from urban Shanghai after substantial economic development (IARC 2010) and the first to report on mortality after household coal use cessation.

Ambient air pollution studies have observed that higher levels of pollutants are associated with increased mortality (Chen et al. 2013; Dockery et al. 1993). Improvement in ambient air pollution has been shown to be associated with reduced mortality over time (Laden et al. 2006; Pope et al. 2009). Retrospective cohort studies in Poland, Czech Republic, and China reported that household air pollution levels were associated with decreased lung function and increased respiratory disease risk in children (Baker et al. 2006; Jedrychowski et al. 2005; Roy et al. 2012). In rural and underdeveloped Xuanwei, China, household coal use was associated with lung cancer incidence/mortality, chronic obstructive pulmonary disease incidence, and pneumonia mortality; however, overall, cardiovascular, or diabetes-related mortality were not studied (Barone-Adesi et al. 2012; Chapman et al. 2005; Lan et al. 2002; Lee et al. 2010; Shen et al. 2009). Previous cohort and case–control studies in Shanghai that assessed coal use and disease risk were focused on stroke and lung cancer incidence rather than overall and cause-specific mortality (Gao 1996; Tao et al. 1991; Zhang et al. 1988). We previously reported in the same population that coal use in homes with poor ventilation was associated with increased lung cancer risk (Kim et al. 2015). In the present analysis, we found a suggestive positive association between coal use and lung cancer mortality. A recent cross-sectional survey observed that solid fuel use in Shanghai was associated with prevalence of hypertension, coronary heart disease, stroke, and diabetes (Lee et al. 2012). Our study adds a prospective cohort study design that assessed all causes of mortality including cardiovascular diseases, cancer and diabetes mortality. However, we did not observe statistically significant associations between coal use and either stroke or diabetes mortality. Last, our study also adds evidence to the hypothesis that coal use cessation leads to a gradual reduction in the relative risk of cardiovascular mortality over time (mortality at > 20 years cessation was similar to never coal users), a similar trend observed for tobacco smoking cessation in the Nurses’ Health Study cohort. The relation between time since quitting and the relative risk for total mortality was examined in the Nurses’ Health Study cohort and the risk of cardiovascular disease and total cancer mortality among former smokers approached the level of never smokers 10–14 years after smoking cessation (Kawachi et al. 1993).

Two of the leading causes of death in China are cancer and cardiovascular disease (He et al. 2005). A sizable proportion of these deaths have been attributed to household combustion of solid fuels (Zhang and Smith 2007). Cardiovascular disease has been shown to account for higher mortality rates than respiratory illness in China (He et al. 2005). In an effort to curb air pollution, several large cities in China have recently banned household coal burning; however, an estimated 10% of ambient PM_2.5_ (PM with diameter ≤ 2.5 μm) derived from cooking with coal in 2010 (Chafe et al. 2014), and an estimated 38% of the population still used coal for cooking in 2009–2010 (Duan et al. 2014) in rural parts of China. Evidence from this study suggests that household coal use could lead to excess deaths years after last exposure among women in Shanghai, although for cardiovascular disease mortality we observed a notable decreasing trend in risk as the time since last use of coal increased. These results underscore the importance of rapidly remediating household coal use in regions where coal use is still common in rural China and other low-middle income nations, to minimize any further adverse health effects, particularly as it relates to mortality from cardiovascular diseases. However, in 2009 about 400 million people still used coal, and about 2.5 billion people used biomass and wood, which warrants further studies (UNDP and WHO 2009).

The primary strengths of this study relate to the prospective nature of the Shanghai Women’s Health Study. This analysis was conducted in a population of middle-class working women in an urban area of China rather than the rural and underdeveloped populations of previous HAP studies (Barone-Adesi et al. 2012; Chapman et al. 2005; Lan et al. 2002; Lee et al. 2010; Shen et al. 2009). This cohort had high participation rates (92.7%) and little loss to follow-up (< 1%), minimizing sources of biases inherent to case–control and retrospective studies such as reverse causation. Last, the population had > 70,000 actively followed participants, and thorough mortality records allowed us to identify 3,808 total deaths. As in most observational studies, exposure misclassification is a concern. Because women were at least 40 years old at enrollment, there may be some historic household conditions or details that were not well remembered by the women during the baseline interview. The vast majority of the subjects in this cohort (97.1%) started using coal when they were < 40 years of age. Any premature deaths that occurred before the minimum cohort eligibility age of 40 years in the general population would be missed, attenuating the observed association between coal use and mortality. The large sample size of this population and the high prevalence of exposure (coal) reduce the likelihood of random error (Jurek et al. 2005). Data on household coal use were available for each woman up until the baseline questionnaire only. Therefore, some degree of exposure misclassification is possible for the duration and time since last use analyses, particularly if subjects continued using coal after completing the baseline interview. However, only 1% of women in this cohort used coal at baseline, and women were highly unlikely to move to a home with coal after the year of the baseline survey. Information on changes in stove quality/modification and other solid fuels was not assessed in the questionnaire, and thus could not be assessed. However, previous evidence has suggested that coal is one of the most hazardous forms of household cooking fuel, and changes in stoves improve health status. For example, stove improvement is likely to reduce the coal combustion by-products and therefore attenuate the observed coal–mortality association (Barone-Adesi et al. 2012). Any changes to improve stoves or other solid fuels would likely attenuate rather than inflate effect estimates. Last, coal from different regions of China may have differing levels of toxicity (Lan et al. 2008) and outdoor/ambient air pollution could affect health status.

## Conclusions

Despite declining current coal use, past coal use in homes of women living in Shanghai was associated with an increased risk of mortality from all causes compared with never use. Women who used coal in their homes for longer periods of time, and women who used coal more recently, had an increased risk of cardiovascular mortality compared with never coal users. Coal burning could be partially responsible for the two leading causes of death in China, cancer and cardiovascular disease, and our findings suggest that effects of coal burning on mortality could persist for years after cessation. These results underscore the immediate importance of remediating coal use in the home where use is still widespread as soon as possible to reduce any further adverse health effects.

## Supplemental Material

(221 KB) PDF
