# Supplementary material for: Cooking Coal Use and All-Cause and Cause-Specific Mortality in a Prospective Cohort Study of Women in Shanghai, China
Source: Environ Health Perspect. 2016 Apr 19;124(9):1384–9. doi: 10.1289/EHP236 (PMC5010399; doi:10.1289/EHP236)
Supplement: (221 KB) PDF [file EHP236.s001.acco.pdf]

Environ Health Perspect
DOI: 10.1289/EHP236

Note to readers with disabilities: *EHP* strives to ensure that all journal content is accessible to all readers. However, some figures and Supplemental Material published in *EHP* articles may not conform to 508 standards due to the complexity of the information being presented. If you need assistance accessing journal content, please contact ehp508@niehs.nih.gov. Our staff will work with you to assess and meet your accessibility needs within 3 working days.

Supplemental Material

Cooking Coal Use and All-Cause and Cause-Specific Mortality in a Prospective Cohort Study of Women in Shanghai, China

Christopher Kim, Wei Jie Seow, Xiao-Ou Shu, Bryan A. Bassig, Nathaniel Rothman, Bingshu Chen, Yong-Bing Xiang, H. Dean Hosgood III, Bu-Tian Ji, Wei Hu, Cuiju Wen, Wong-Ho Chow, Qiuyin Cai, Gong Yang, Yu-Tang Gao, Wei Zheng, and Qing Lan

Table of Contents

Table S1. Risk of all-cause and cause-specific mortality by ever usage of coal in Shanghai Women's Health Study, stratified by family annual income

Table S2. Risk of all-cause and cause-specific mortality by ever usage of coal in Shanghai Women's Health Study, stratified by education

Table S3. Risk of all-cause and cause-specific mortality by ever usage of coal in Shanghai Women's Health Study, stratified by kitchen ventilation

Table S1. Risk of all-cause and cause-specific mortality by ever usage of coal in Shanghai Women's Health Study, stratified by family annual income

		≥20,000 yuan		<20,000 yuan		
	Never used coal	Ever used coal		Ever used coal		
Cause of death (ICD-9)	HR(95% CI)[a]	Deaths[b]	HR(95% CI)[a]	Deaths[b]	HR(95% CI)[a]	Interaction *P*
All cause	1.00 (ref)	727	1.17 (1.04, 1.32)	1772	1.10 (1.01, 1.20)	0.46
Cancer (140-208)	1.00 (ref)	345	1.12 (0.95, 1.32)	699	1.17 (1.01 ,1.34)	0.70
Lung cancer (162)	1.00 (ref)	73	1.18 (0.81, 1.70)	141	1.20 (0.88, 1.63)	0.52
Cardiovascular (390-459)	1.00 (ref)	153	1.40 (1.07, 1.85)	430	1.10 (0.92, 1.32)	0.19
Ischemic heart disease (410-414)	1.00 (ref)	42	2.31 (1.25, 4.29)	91	1.33 (0.88, 2.03)	0.24
Myocardial infarction (410)	1.00 (ref)	31	3.79 (1.67, 8.60)	60	1.25 (0.74, 2.10)	0.08
Stroke (430-438)	1.00 (ref)	88	1.24 (0.87, 1.77)	261	0.94 (0.75, 1.17)	0.19
Ischemic stroke (434)	1.00 (ref)	34	1.05 (0.60, 1.84)	111	1.11 (0.78, 1.57)	0.88
Hemorrhagic stroke (430-432)	1.00 (ref)	46	1.31 (0.81, 2.14)	119	0.77 (0.56, 1.06)	0.12
Gastrointestinal (520-579)	1.00 (ref)	18	1.35 (0.63, 2.88)	37	1.24 (0.64, 2.42)	0.81
Kidney disease (580-593)	1.00 (ref)	13	1.19 (0.49, 2.92)	33	1.07 (0.57, 2.00)	0.66
Diabetes (250)	1.00 (ref)	11	0.98 (0.38, 2.51)	55	1.50 (0.82, 2.73)	0.26

[a]Adjusted for age, smoking status, environmental tobacco smoke, occupation, education, shift work, BMI, hormone therapy, family income, parity, alcohol drinking, marital status, caloric intake, physical activity, stove ventilation.
[b]Women were excluded from analyses of specific causes of death if they reported a history of corresponding diseases at baseline.

Table S2. Risk of all-cause and cause-specific mortality by ever usage of coal in Shanghai Women's Health Study, stratified by education

		Middle school and above		Elementary school and below		
	Never used coal	Ever used coal		Ever used coal		
Cause of death (ICD 9)	HR(95% CI)[a]	Deaths[b]	HR(95% CI)[a]	Deaths[b]	HR(95% CI)[a]	Interaction *P*
All cause	1.00 (ref)	1014	1.21 (1.09,1.34)	1486	1.06 (0.96, 1.17)	0.10
Lung cancer (162)	1.00 (ref)	102	1.38 (1.00 1.91)	113	1.03 (0.73, 1.46)	0.43
Cancer (140-208)	1.00 (ref)	530	1.25 (1.08, 1.44)	515	1.03 (0.87, 1.21)	0.13
Cardiovascular (390-459)	1.00 (ref)	188	1.42 (1.11, 1.81)	395	1.09 (0.90, 1.32)	0.24
Ischemic heart disease (410-414)	1.00 (ref)	36	1.86 (1.02, 3.40)	97	1.56 (1.02, 2.38)	0.76
Myocardial infarction (410)	1.00 (ref)	27	1.86 (0.93, 3.72)	64	1.88 (1.07, 3.32)	0.84
Stroke (430-438)	1.00 (ref)	118	1.23 (0.90, 1.66)	231	0.92 (0.72, 1.17)	0.15
Ischemic stroke (434)	1.00 (ref)	45	1.31 (0.80, 2.15)	100	0.99 (0.69, 1.43)	0.29
Hemorrhagic stroke (430-432)	1.00 (ref)	65	1.17 (0.78, 1.74)	100	0.77 (0.54, 1.09)	0.15
Gastrointestinal (520-579)	1.00 (ref)	23	1.80 (0.85, 3.83)	32	1.00 (0.52, 1.93)	0.24
Kidney disease (580-593)	1.00 (ref)	18	1.32 (0.62, 2.82	28	0.91 (0.46, 1.81)	0.50
Diabetes (250)	1.00 (ref)	21	1.57 (0.66, 3.72)	45	1.34 (0.73, 2.47)	0.42

[a]Adjusted for age, smoking status, environmental tobacco smoke, occupation, shift work, BMI, hormone therapy, family income, parity, alcohol drinking, marital status, caloric intake, physical activity, stove ventilation.
[b]Women were excluded from analyses of specific causes of death if they reported a history of corresponding diseases at baseline.

Table S3. Risk of all-cause and cause-specific mortality by ever usage of coal in Shanghai Women's Health Study, stratified by kitchen ventilation

Coal Use	Never used coal	Always good ventilation: Ever used coal		Always fair/poor ventilation: Ever used coal		Interaction *P*
Cause of death (ICD 9)	HR(95% CI)[a]	Deaths[b]	HR(95% CI)[a]	Deaths[b]	HR(95% CI)[a]	
All cause	1.00 (ref)	355	1.03 (0.90, 1.19)	505	1.16 (1.01, 1.33)	0.92
Cancer (140-208)	1.00 (ref)	169	1.20 (0.98, 1.47)	199	1.01 (0.82, 1.25)	0.75
Lung cancer (162)	1.00 (ref)	34	1.08 (0.70, 1.67)	35	1.13 (0.67, 1.91)	0.16
Cardiovascular (390-459)	1.00 (ref)	81	1.00 (0.75, 1.33)	120	1.15 (0.86, 1.54)	0.71
Ischemic heart disease (410-414)	1.00 (ref)	23	1.79 (0.97, 3.30)	30	2.04 (1.01, 4.12)	0.29
Myocardial infarction (410)	1.00 (ref)	16	2.39 (1.09, 5.26)	21	2.71 (1.06, 6.89)	0.60
Stroke (430-438)	1.00 (ref)	35	0.70 (0.46, 1.04)	68	0.90 (0.63, 1.30)	0.59
Ischemic stroke (434)	1.00 (ref)	11	0.47 (0.24, 0.93)	32	1.09 (0.63, 1.91)	0.53
Hemorrhagic stroke (430-432)	1.00 (ref)	21	0.89 (0.52, 1.53)	30	0.72 (0.43, 1.19)	0.53
Gastrointestinal (520-579)	1.00 (ref)	6	0.86 (0.31, 2.35)	9	1.71 (0.49, 6.00)	0.32
Kidney disease (580-593)	1.00 (ref)	8	0.84 (0.34, 2.07)	6	0.95 (0.25, 3.60)	0.99
Diabetes (250)	1.00 (ref)	7	1.74 (0.56, 5.37)	16	2.17 (0.84, 5.62)	0.066

[a]Adjusted for age, smoking status, environmental tobacco smoke, occupation, education, shift work, BMI, hormone therapy, family income, parity, alcohol drinking, marital status, caloric intake, physical activity, stove ventilation.

[b]Women were excluded from analyses of specific causes of death if they reported a history of corresponding diseases at baseline.
